# Modification of Deoxynivalenol by a Fungal Laccase Paired with Redox Mediator TEMPO

**DOI:** 10.3390/toxins14080548

**Published:** 2022-08-11

**Authors:** Hina Shanakhat, Susan P. McCormick, Mark Busman, Joseph O. Rich, Matthew G. Bakker

**Affiliations:** 1 Department of Agricultural Sciences, University of Naples Federico II, 80138 Naples, Italy; 2 National Center for Agricultural Utilization Research, Agricultural Research Service, United States Department of Agriculture, Peoria, IL 61604, USA; 3 Plains Area Office, Agricultural Research Service, United States Department of Agriculture, Fort Collins, CO 80526, USA; 4 Department of Microbiology, University of Manitoba, Winnipeg, MB R3T 2N2, Canada

**Keywords:** deoxynivalenol, laccase, mycotoxin, trichothecene

## Abstract

Mycotoxins such as deoxynivalenol introduce a health risk to the food supply and are costly to manage or avoid. Technologies for reducing or eliminating the toxicity of deoxynivalenol could be useful in a variety of processes, such as in preserving the value as animal feed of byproducts of ethanol production. We characterized transformation products of deoxynivalenol that were formed by the combination of a fungal laccase paired with the chemical mediator 2,2,6,6-tetramethylpiperidine-N-oxyl (TEMPO), using chromatography, mass spectrometry, and nuclear magnetic resonance spectroscopy. Alcohol groups at the C3 and C15 positions of deoxynivalenol were oxidized to ketones, and the chemical mediator became covalently linked to the C4 position. Conditions experienced during gas chromatography led to the dissociation of TEMPO, forming 3,15-diketodeoxynivalenol. Understanding the range of possible modifications to deoxynivalenol and other trichothecenes is a necessary step toward effective remediation of contaminated grain.

## 1. Introduction

Many fungal species produce bioactive secondary metabolites, some of which are toxic to humans or animals and are categorized as mycotoxins. Mycotoxins frequently contaminate crops, and it has been estimated that globally, 25% of food crops could be affected by mycotoxins each year [1]. As one example, annual losses associated with mycotoxins are valued at up to 1.6 billion US dollars for the corn industry in the United States alone [2,3], and these costs do not include negative impacts on human health. The mycotoxins of largest current concern include aflatoxins, deoxynivalenol (DON), zearalenone, fumonisins, patulin, and ochratoxin.

DON, also known as vomitoxin, belongs to the trichothecenes, which are a group of sesquiterpenoid secondary metabolites that are produced by several different species of *Fusarium* [4] as well as other fungi. In wheat and barley, DON accumulation is associated with the disease fusarium head blight [5]. The same pathogen causes gibberella ear rot in corn. Together, these diseases lead to tremendous loss in yield and quality of cereal grains and ultimately pose a great threat to food safety and public health [6]. Exposure to DON causes diarrhea, abdominal pain, vomiting, skin irritation [7] and immunosuppressive effects [8], depending on the dose and duration of exposure.

To protect against mycotoxins requires control strategies at every stage of the supply chain, from in-field strategies during crop production through to proper storage, increasingly strategies are sought for removing or remediating mycotoxins during processing into food or feed products [9,10]. Post-harvest mitigation of mycotoxin contamination can include physical, chemical, biological, and enzymatic strategies. Physical removal of mycotoxins can be accomplished by a variety of methods before and during food processing, such as grain sorting, dehulling, irradiation, or the use of higher temperatures for baking and frying [11]. Chemical controls include the use of oxidizing agents, reducing agents, acids or bases for the purpose of degrading mycotoxins. Ultimately, such chemical detoxification strategies require regulatory approvals because of potential negative effects on food quality as a result of chemical residues [12].

Biological detoxification is an emerging approach that involves the use of different microorganisms, or their enzymes, for biotransformation of mycotoxins into less toxic compounds [13,14,15,16,17]. One challenge for enzymatic degradation is that the catalytic specificity of enzymes may limit detoxification to a narrow range of mycotoxin chemical structures. However, some enzymes, such as laccases, exert their activities through less specific mechanisms and may possess a broader spectrum of activity [18,19,20], or even have utility for the simultaneous degradation of multiple mycotoxins. The laccases are a family of multicopper oxidases and have been shown to promote oxidative reactions with aromatic amines, phenols, and other non-phenolic compounds by catalyzing the reduction of molecular oxygen into water [20,21]. In some cases, interaction of laccases and substrates may be indirect, occurring via the formation of reactive intermediates with strong oxidoreductive properties. Thus, the activity of laccases can often be enhanced by combining the enzyme with a redox mediator [22]. Among the compounds that have been widely used as redox mediators in biocatalytic processes are 2,2,6,6-tetramethylpiperidine-N-oxyl (TEMPO), 1-hydroxybenzotriazole, and 2,2-azino-bis-[3-ethylbenzo-thiazolin-sulfonate] [23].

Laccases are currently being used in food industries (i.e., baking, fermentation, dairy processing) to improve food techno-functional properties, modify food sensory parameters, or improve product shelf-life [24]. This suggests that it may be feasible to integrate laccase treatment for mycotoxin remediation into food processing workflows. Moreover, laccases have also been applied in other industries for bioremediation [25], chemical synthesis [26], and pharmaceutical and cosmetic manufacturing [27]. Laccases have been used successfully to chemically modify some mycotoxins, such as certain aflatoxins and zearalenone [16,28,29].

The objective of this study was to determine whether laccases could be used to chemically modify DON. Because we found evidence of such activity, we developed a supporting objective to characterize the nature of the chemical changes that were made to DON.

## 2. Results

### 2.1. Preliminary Screening of Laccase Enzymes and Chemical Mediators

**Figure A1:**
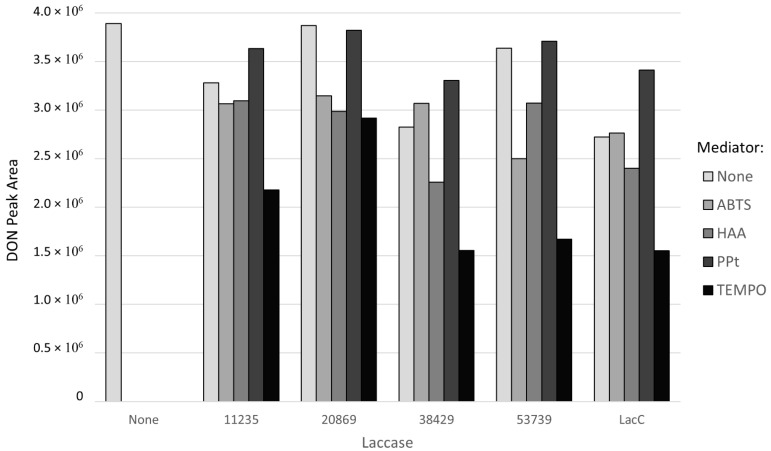
Combinations of laccase enzyme + chemical mediator exhibit a range of DON-transforming activities. All reactions were initiated with the same quantity of DON. DON content was assessed by GC/MS after incubation of the reaction mixture. Tested laccases included crude preparations from *Trametes versicolor* ATCC 11235 (’11235’) or from *Trametes versicolor* ATCC 20869 (‘20869’), as well as commercial products: 38429 (Fluka; derived from *Trametes versicolor*), 53739 (Fluka; derived from *Trametes versicolor*), and LacC (ASA Chemical Group; derived from *Trametes* sp.). Four mediators were tested: 2,2-azino-bis(3-ethylbenzothiazoline-6-sulfonic acid) diammonium salt (ABTS), 3-hydroxyanthranilic acid (HAA), phenolphthalein (PPt), and 2,2,6,6-tetramethyl-1-piperidinyloxy, free radical (TEMPO). Although n = 1, the effect of interest was validated with further experiments.

**Figure A2:**
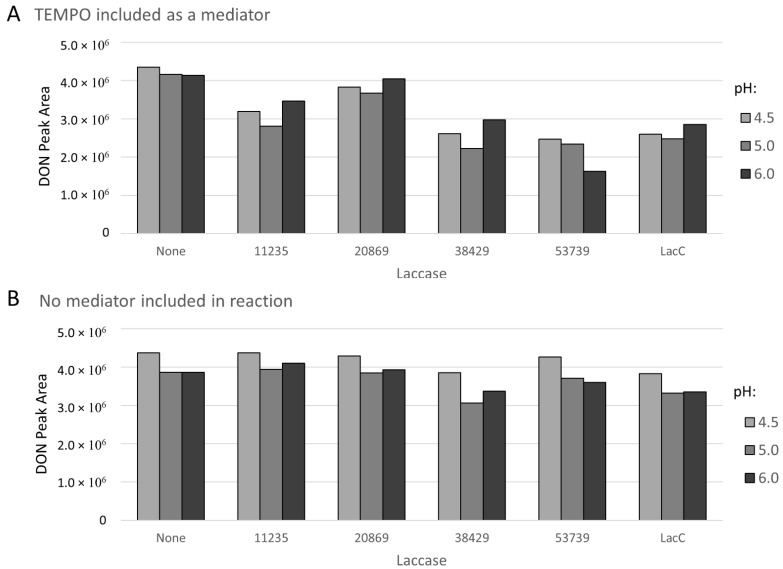
DON-transforming activity of a variety of laccase enzymes across several pH levels, when (**A**) paired with the redox mediator 2,2,6,6-tetramethyl-1-piperidinyloxy, free radical (TEMPO), or (**B**) not paired with any mediator. All reactions were initiated with the same quantity of DON. DON content was assessed by GC/MS after incubation of the reaction mixture. Tested laccases included crude preparations from *Trametes versicolor* ATCC 11235 (‘11235’) or from *Trametes versicolor* ATCC 20869 (‘20869’), as well as commercial products: 38429 (Fluka; derived from *Trametes versicolor*), 53739 (Fluka; derived from *Trametes versicolor*), and LacC (ASA Chemical Group; derived from *Trametes* sp.). Although n = 1, the effect of interest was validated with further experiments.

Preliminary screens of laccase enzymes and chemical mediators suggested that incubation at 28 °C in McIlvaine’s buffer at pH 5.0 provided suitable conditions for laccase activity (data not shown). Gas chromatography coupled with mass spectrometry (GC/MS) was used to screen different laccases and different mediators in a combinatorial fashion. Several combinations of laccases and mediators caused in a reduction in the amount of DON remaining in the reaction mixture. Among the mediators tested, addition of TEMPO resulted in the greatest loss of DON with three of the five laccases tested, reaching as low as 40% of the input DON remaining after incubation (Figure A1). Based on these initial screens, we selected laccase 53739 and the redox mediator TEMPO for further study. Reduction of DON peak area was less in the absence than in the presence of the mediator; for instance, in the absence of TEMPO, 93–98% (variation among buffer pH levels) of the input DON remained after incubation with laccase 53739, while in the presence of TEMPO, only 39–57% of the input DON remained after incubation with the same laccase (Figure A2). However, we did not determine whether there were statistically significant rates of conversion under these conditions and did not pursue further any potential laccase activity in the absence of TEMPO.

### 2.2. Appearance of New Gas Chromatography/Mass Spectrometry Peaks

**Figure 1:**
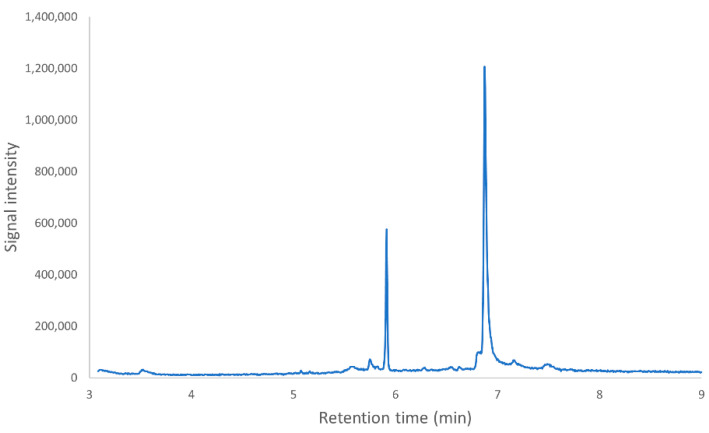
Total ion chromatogram of reaction products after incubation of DON with laccase 53739 + TEMPO. The expected peak for DON at 6.29 min is missing, while two new peaks have appeared at retention times 5.91 and 6.92 min.

Reaction mixtures containing DON, laccase and TEMPO were optimized for the complete loss of the GC/MS peak for DON (retention time 6.29 min) by reducing the input concentration of DON 10-fold, to 0.075 mg/mL. After the reaction, two new peaks were present, at 5.91 min and at 6.92 min (Figure 1).

DON has a molecular ion at *m*/*z* = 296, whereas the two new GC/MS peaks had molecular ions at *m*/*z* = 294 (6.92 min) and *m*/*z* = 292 (5.91 min). These decreases in steps of two atomic mass units for the molecular ion are consistent with the successive oxidations of hydroxyl groups to keto groups.

**Figure 2:**
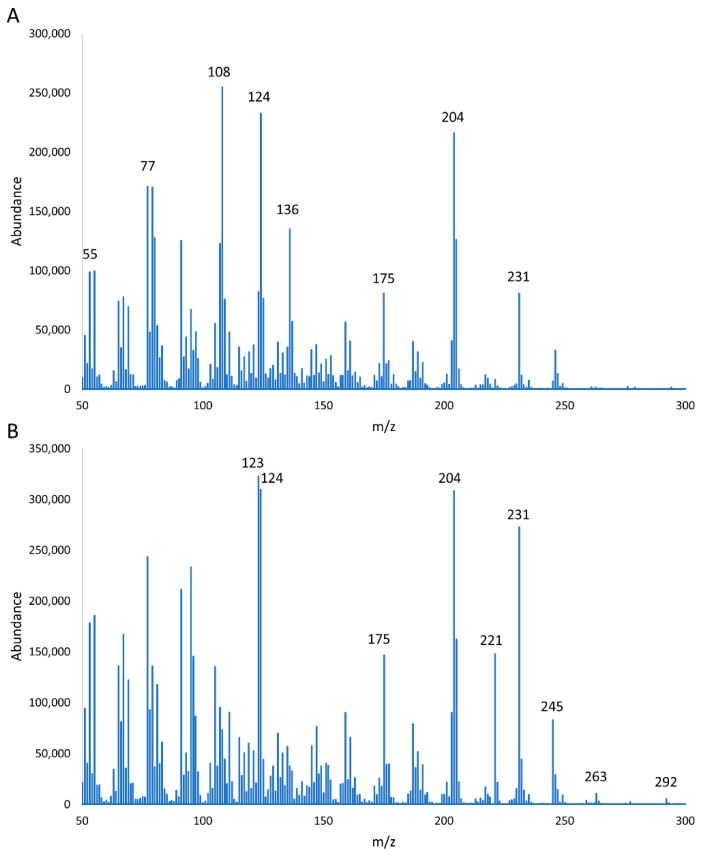
Mass spectra of DON derivatives: (**A**) the GC/MS peak at 6.92 min; (**B**) the GS/MS peak at 5.91 min.

Gas chromatography/mass spectrometry data were very good matches to two previously reported derivatives of DON: 3-keto DON and 3,15-diketo DON [30]. Specifically, ions at *m*/*z* = 55, 77, 108, 124, 136, 175, 204, and 231 observed for the 6.92 min peak matched ions reported for 3-keto DON, while ions at *m*/*z* = 123, 124, 175, 204, 221, 231, 245, 263, and 292 for the 5.91 min peak matched ions reported for 3,15-diketo DON (Figure 2) [30].

To further validate the positions of the molecule that were oxidized, we repeated the enzyme assays using two trichothecene variants that are each substituted with an acetyl group in place of a hydroxyl group (i.e., 15-acetyl-DON and 3-acetyl-DON). As anticipated, 15-acetyl-DON, having an acetyl group at atom position C15, formed a single product, with a mass spectrum consistent with 3-keto-15-acetyl-DON. Similarly, 3-acetyl-DON, having an acetyl group at atom position C3, formed a single product, with a mass spectrum consistent with 15-keto-3-acetyl-DON (data not shown).

In sum, an initial identification by gas chromatography and mass spectrometry suggested a stepwise transformation of DON into 3-keto-DON and then to 3,15-diketo-DON.

### 2.3. Liquid Chromatography/Mass Spectrometry

**Figure 3:**
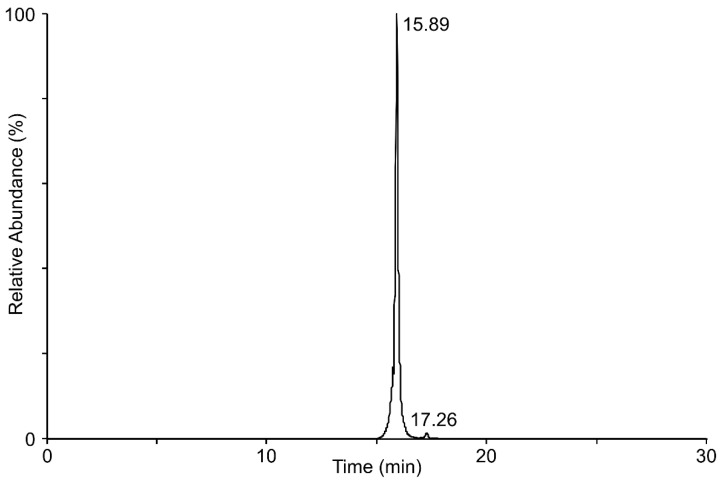
LC/MS chromatogram showing the presence of two transformation products after incubating DON with laccase 53739 + TEMPO: a major peak at a retention time of 15.89 min, and a minor peak at a retention time of 17.26 min.

To view these transformation products under different conditions, we also screened reaction products using liquid chromatography/mass spectrometry. Analysis of reaction mixtures (DON + laccase + TEMPO) found two transformation products were detected at 15.89 min and 17.26 min (Figure 3).

**Figure 4:**
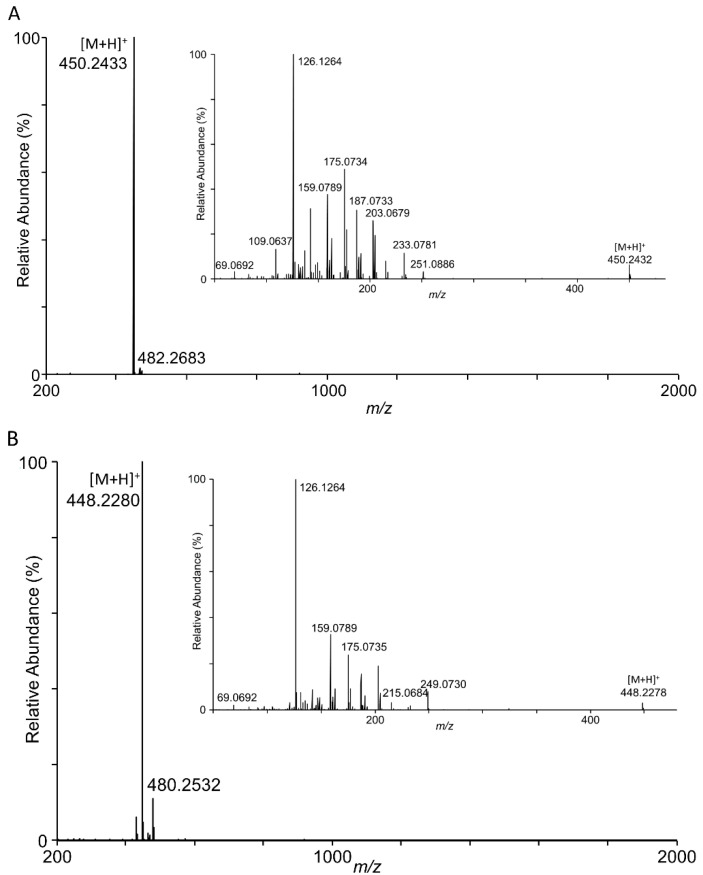
LC/MS analyses of peaks at (**A**) retention time of 15.89 min; (**B**) retention time of 17.26 min. In each panel, the inset mass spectrum reflects electrospray ionization tandem mass spectrometry (ESI-MS/MS) of the primary ion.

The molecular ions for the two transformation products again differed by two atomic mass units (in positive mode, *m*/*z* for [M + H]^+^ = 450 for the peak at 15.89 min and *m*/*z* for [M + H]^+^ = 448 for the peak at 17.26 min). However, these ions were larger than expected and did not match directly with the expected identity of the compounds as 3-keto-DON and 3,15-diketo-DON. The main product (retention time 15.89 min, *m*/*z* = 450) was consistent with a TEMPO conjugate of 3-keto-DON; i.e., *m*/*z* = (156 + 294). The minor product (retention time 17.26 min, *m*/*z* = 448) was consistent with a TEMPO conjugate of 3,15-diketo-DON; i.e., *m*/*z* = (156 + 292) (Figure 4). Fragmentation of both transformation products by electrospray ionization tandem mass spectrometry (ESI-MS/MS) was performed for the better understanding of the transformation products (Figure 4).

When viewed in negative mode, the compound eluting at 15.89 min yielded a mass spectrum with dominant ions at *m*/*z* = 508 and 308. Tandem mass spectrometry demonstrated that the *m*/*z* = 308 ion was a fragmentation product of the *m*/*z* = 508 ion (ESI-MS/MS; data not shown). When a DON standard was run under these LC/MS conditions, we also observed DON at a larger than expected mass (*m*/*z* = 355), which when fragmented, produced an ion that was consistent with liberation of an acetoxy group (*m*/*z* = 59), likely picked up from the LC solvent. The observed *m*/*z* = 508 would then be consistent with 3-keto-DON ([M + H]^+^, m.w. = 293) associated with an acetoxy group (m.w. = 59) plus the chemical mediator TEMPO (m.w. = 156).

**Figure 5:**
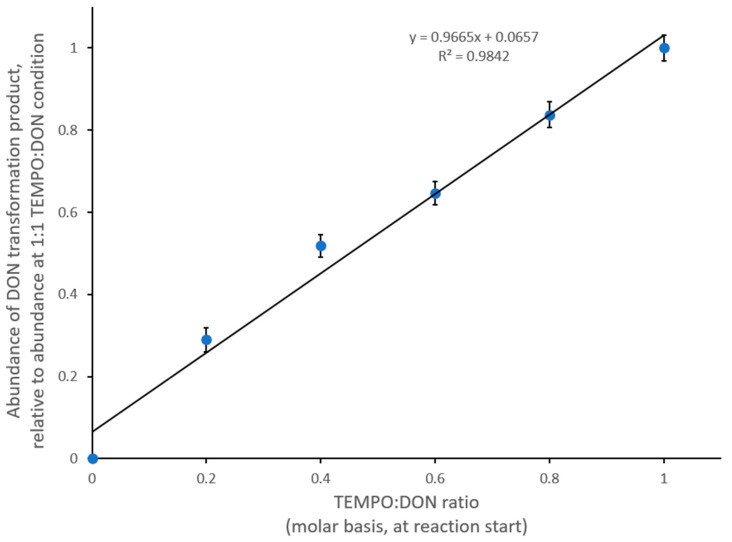
Abundance of the DON transformation product (LC/MS in positive mode, intensity for the peak at 15.89 min, with *m*/*z* = 450) depends on the availability of TEMPO in the reaction.

To confirm the possible role of TEMPO as a substrate in this reaction, we varied the molar ratio of TEMPO:DON from 0:1–1:1. It was interesting to note that the conversion of DON increased linearly with increasing TEMPO concentrations (Figure 5). This suggests that TEMPO is a substrate in the reaction, rather than functioning in the intended capacity as a mediator in a catalytic cycle, and is consumed in equimolar amounts to DON. Therefore, it is possible that the conjugation of DON and TEMPO occurs as part of the reaction.

### 2.4. Nuclear Magnetic Resonance Spectroscopy

**Figure 6:**
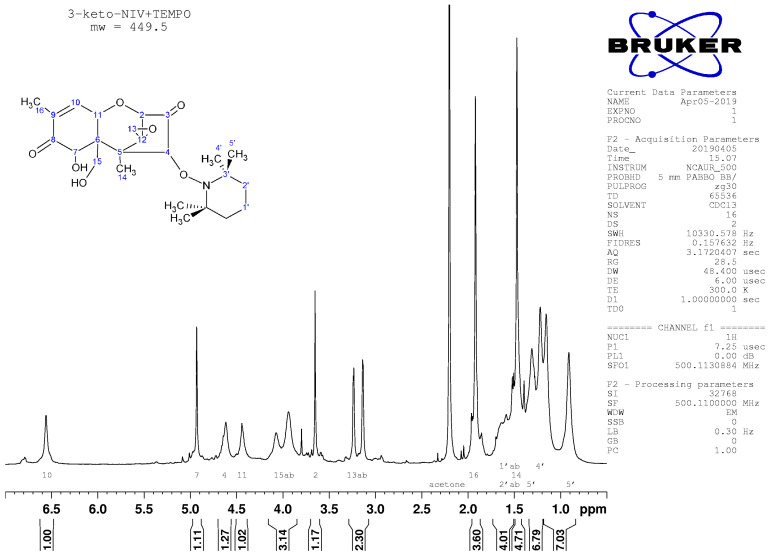
^1^H NMR spectrum for transformation product 1, with assignments.

**Figure 7:**
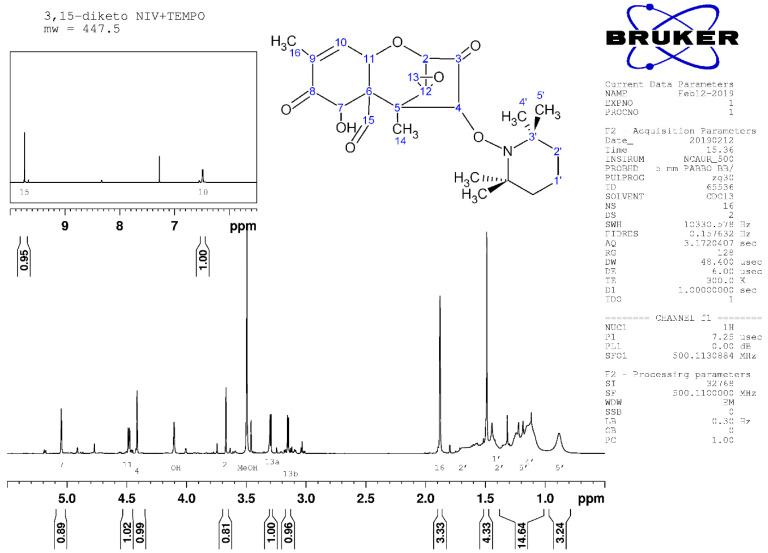
^1^H NMR spectrum for transformation product 2, with assignments.

**Figure A3:**
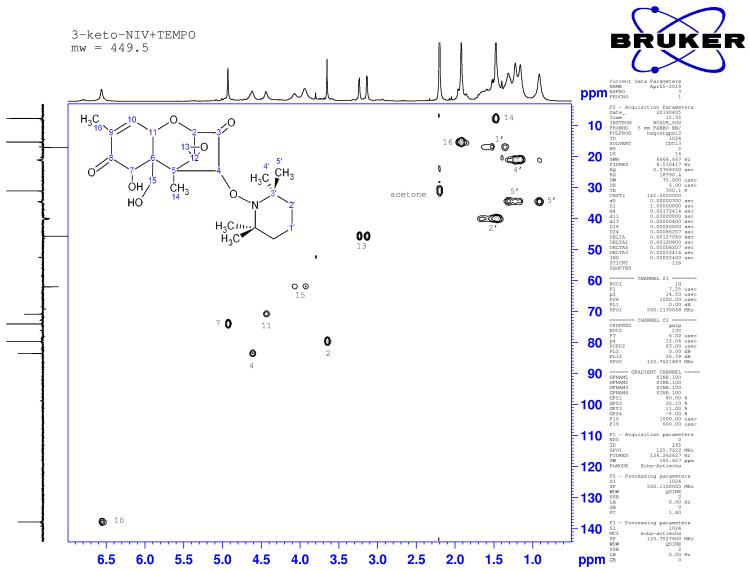
Heteronuclear single quantum coherence NMR spectrum for transformation product 1, with assignments.

**Figure A4:**
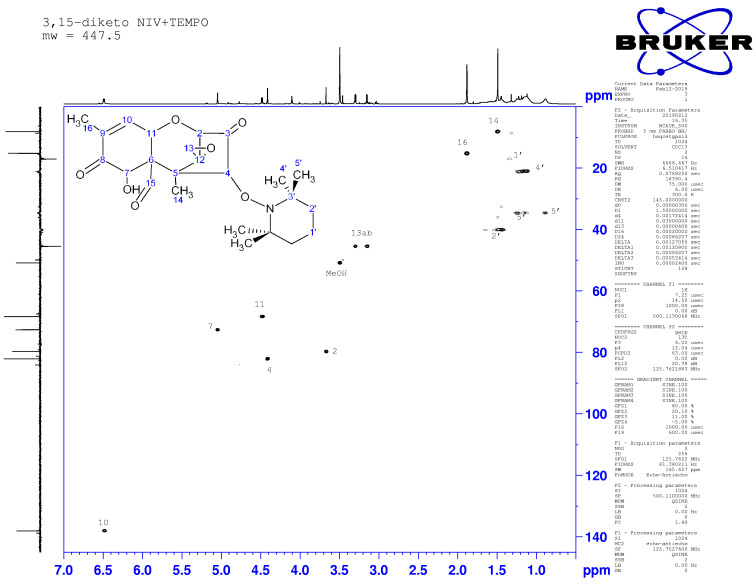
Heteronuclear single quantum coherence NMR spectrum for transformation product 2, with assignments.

Nuclear magnetic resonance spectroscopy confirmed the LC/MS indication that the transformation products were 3-keto,4-TEMPO-DON and 3,15-diketo,4-TEMPO-DON. In particular, a chemical shift for C3 was absent in ^1^H NMR for both compounds (Figure 6 and Figure 7), supporting the absence of protons at this position in the oxidized products. That C4 is substituted (i.e., with TEMPO) is evident in both products by the presence of a single, rather than doublet, signal for this position in ^1^H NMR (Figure 6 and Figure 7). Diastereotopic protons at C15 were evident in transformation product 1 (Figure 6 and Figure A3), but were absent in transformation product 2 (Figure 7 and Figure A4), indicating oxidation and loss of a proton from C15 in the progression from the first to the second product. In the first transformation product, broad peaks for protons at C4, C11, and C15 (Figure 6) are attributable to hydrogen bonding between the OH at C15 and the N in TEMPO. Along with the oxidation of C15 to form the second transformation product, such hydrogen bonding would be eliminated and peaks for C4 and C11 become much sharper (Figure 7).

**Figure 8:**
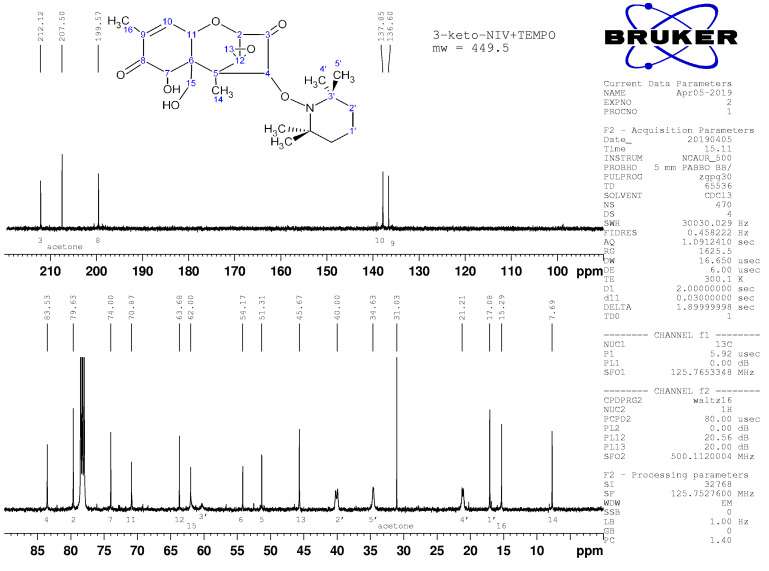
^13^C NMR spectrum for transformation product 1, with assignments.

**Figure 9:**
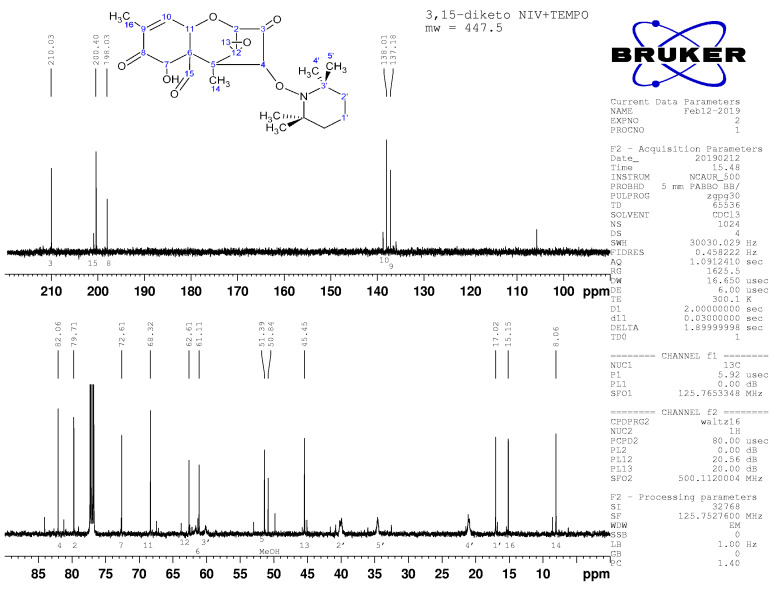
^13^C NMR spectrum for transformation product 2, with assignments.

The oxidation of C15 to form 3,15-diketo,4-TEMPO-DON is also supported by impacts on the peaks for C6 and C15 in ^13^C NMR (Figure 8 and Figure 9). Additional peaks beyond those attributable to DON were clearly present and readily assignable to TEMPO (Figure 8, Figure 9, Figure A3 and Figure A4). Broad peaks in the ^13^C NMR spectra are attributable to the N in TEMPO.

## 3. Discussion

While we have a very detailed understanding of the step-by-step synthesis of trichothecenes (see Figure 1 in [4]), exploration of the various products into which other microbes may convert trichothecenes has just begun [31]. In this work, the combination of laccase 53739 from *Trametes versicolor* plus the chemical mediator TEMPO was found to oxidize DON at the C3 and (less efficiently) C15 positions, and to covalently link a redox-active chemical mediator to the C4 position. Linkage of TEMPO to C4 is somewhat unstable, as indicated by the dissociation of this moiety under GC/MS conditions. To our knowledge, 3,15-diketo-DON has not been reported as a product of enzymatic transformation. However, it has been reported with a suite of derivatives generated chemically from DON [30].

The C3 position of deoxynivalenol is a frequent site of transformation [32]. For example, a dehydrogenase that oxidizes position C3 from an alcohol to a ketone group has been described [33], and 3-ketoDON can be reduced in stereospecific fashion to yield 3-epi-DON [34,35]. The C3 position is also targeted by plants in a glycosylation reaction that reduces toxicity [36,37]. Both the C3 and C15 positions have also been implicated in the formation of DON-sulfates [38]. Clearly, there are some characteristics of the C3 and, to a lesser extent the C15, positions of the molecule that make them more available to modification. Notably, however, enzymatic transformation of other positions has been reported, such as a cytochrome P450-mediated oxidation at the C16 position [39]. We expect that additional discovery efforts will reveal enzymes with the ability to transform DON in diverse ways.

The aim of characterizing transformations to the DON molecule is generally to identify mechanisms by which the toxicity of the molecule can be reduced. For instance, it has previously been shown that epimerization at the C3 position of DON reduces toxicity [40] and enhancing the ability to glycosylate DON improves plant resistance toward fusarium head blight [41], which is an indication of reduced toxicity in the glycosylated product. The toxicology of DON transformation products has recently been reviewed [42].

TEMPO and its derivatives have been well-studied as mediators in many biotransformation reactions catalyzed by laccases from a variety of microbial sources, including fungi such as *Trametes versicolor* [43,44], and TEMPO was part of the most effective DON conversions in our laccase assays. Since the first report of a mediated laccase-catalyzed reaction [45], more than 100 compounds have been tested as potential mediators. However, the rational a priori choice of a mediator for a given reaction has been difficult, and empirical testing remains necessary to identify suitable laccase–mediator combinations for particular transformations of interest. Unusually, the observed product structures in our system indicate that TEMPO is a substrate in the reaction, rather than a mediator of a catalytic cycle. However, the mediator did not react in this way with DON in the absence of laccase (Figure 2). It would be beneficial to screen other oxidizable mediators in order to identify a mediator that functions strictly catalytically, rather than being incorporated into the transformation products.

The identification of a system for the oxidization of DON to less toxic forms would be a significant step towards the effective management of DON. For instance, there is great potential for applications of technologies for detoxification of DON and other mycotoxins in the animal feed industry [46] or to preserve the value of byproducts from ethanol production. The ability to use commercially available enzymes to chemically modify DON suggests the potential for rapid implementation in industrial applications. However, the present work has not established whether the transformation products described here are in fact less toxic than DON. For instance, it has recently been shown that in some plant models, the toxicity of 3-keto-DON is equivalent to the toxicity of DON itself [47]. Neither has this work tested the feasibility of the observed transformation in the presence of grain, foodstuffs, or other matrices which could offer alternative sites that may be more favorable for oxidation compared to the C3 and C15 hydroxyl groups of the DON molecule. That is to say, in more complicated chemical contexts, as would be present in real-world scenarios in which one might want to remediate grain that has become contaminated with mycotoxins, there would likely be multiple off-target sites upon which laccase + mediator may act; further development is needed to understand the action and efficacy of this system for chemically modifying DON under more realistic scenarios.

## 4. Materials and Methods

### 4.1. Development of the Laccase-Mediator System

Preliminary assays were used to test laccase activity in transforming known substrates in order to optimize assay pH, temperature, and duration of incubation. Reactions were performed in a combinatorial fashion with five laccase enzymes from different suppliers or strains of the fungus *Trametes* and four chemical mediators. Tested laccases included crude preparations from *Trametes versicolor* ATCC 11235 (‘11235’) or from *Trametes versicolor* ATCC 20869 (‘20869’), as well as commercial products: 38429 (sourced from Fluka; produced by *Trametes versicolor*), 53739 (sourced from Fluka; produced by *Trametes versicolor*), and LacC (sourced from ASA; produced by *Trametes* sp.). Four mediators were tested: 2,2-azino-bis(3-ethylbenzothiazoline-6-sulfonic acid) diammonium salt (ABTS; sourced from Fluka), 3-hydroxyanthranilic acid (HAA; TCI), phenolphthalein (PPt; Sigma-Aldrich, St. Louis, MO, USA), and 2,2,6,6-tetramethyl-1-piperidinyloxy, free radical (TEMPO; Aldrich). Assays for testing different reaction conditions were performed in triplicate 100 or 200 µL volumes in 96-well polystyrene plates. Assays for determination of optimal pH consisted of laccase at 10 mg/mL, methanol at 10% of the total volume, 0.02 mM syringaldazine (SAFC, St. Louis, MO, USA), and McIlvaine buffer (sodium phosphate dibasic + citric acid monohydrate) constituted to yield pH values ranging from 2.5 to 8.0 (for example, to prepare McIlvaine buffer at pH = 5.0, final concentration of sodium phosphate dibasic is 0.10 M and final concentration of citric acid monohydrate is 0.049 M; for pH = 6.0, final concentration of sodium phosphate dibasic is 0.13 M and final concentration of citric acid monohydrate is 0.037 M). Laccase activity was measured using a plate reader (Molecular Devices, Sunnyvale, CA, USA) at 530 nm for 5 min, with oxidation of syringaldazine causing an increase in absorbance. Assays for determination of optimum incubation temperature and duration consisted of 1 mM ABTS dissolved in 180 µL McIlvaine buffer at the selected pH, with 20 µL of enzyme solution. Assays were incubated across a temperature range of 25–80 °C and oxidation of ABTS was assessed as an increase in absorbance at 420 nm [48].

Assays for laccase-mediated transformation of DON were performed in 1.5 mL microcentrifuge tubes. Based on results of preliminary trials, reaction mixtures were set up by adding 30 µL of DON solution (5 mg/mL in water, filter-sterilized), followed by 150 µL of TEMPO free radical (6.5 mM; Aldrich, Steinheim, Germany) dissolved in McIlvaine buffer at pH 5.0, and 20 µL of laccase 53739 (10 mg/mL). Thus, final reaction concentrations were 0.75 mg/mL DON, 1 mg/mL laccase, 4.875 mM TEMPO, 0.075 M sodium phosphate dibasic, 0.0364 M citric acid. In some cases, the amount of DON in the assay was varied (e.g., reduced input quantity by up to 10-fold to achieve complete conversion), or 15-acetyl-deoxynivalenol (15-ADON) or 3-acetyl-deoxynivalenol (3-ADON) was substituted for DON. Assays were incubated at 28 °C, 200 rpm for 72 h. Control samples containing DON but no laccase or no mediator were incubated under the same conditions. Reactions were stopped after 24, 48, or 72 h of incubation, by the addition of an equal volume (200 µL) of acetonitrile. The reaction mixtures were transferred to 1-dram vials and were dried under N_2_ gas at 55 °C.

### 4.2. Characterization of Transformation Products

The dried residues of reaction mixtures were redissolved in 500 µL methanol. Gas chromatography/mass spectrometry (GC/MS) analyses were performed with an Agilent (Santa Clara, CA, USA) 6890 gas chromatograph fitted with an HP-5MS column (30 m length × 0.25 mm internal diameter × 0.25 μm film thickness) and paired with an Agilent 5973 mass spectrometer. The carrier gas was helium with 20:1 split ratio and a flow rate of 20 mL/min. Samples were injected at 150 °C, the temperature was held for 1 min and then the column was heated to 280 °C at a rate of 30 °C/min and held for 3.7 min. Under these conditions, DON was detected at 6.2 min.

Liquid chromatography/mass spectrometry (LC/MS): the dried residues of reaction mixtures were redissolved in 500 µL of methanol:water (86:14 by volume). Analyses were performed with a Dionex UltiMate 3000 UPLC attached to a Thermo QExactive MS. Compounds were separated on a Phenomenex Kinetex F5 column (150 mm length × 2.1 mm internal diameter, 1.7 µm particle size, 100 Å pore diameter) with gradient elution using water/methanol (0.2 mL/min, 15–95% methanol over 15 min) containing 5 mM ammonium acetate. The MS was operated utilizing electrospray ionization and set to detect ions in full-scan high-resolution mode (resolution = 70,000) over the mass range 100–1000. Each sample was run with the MS set to detect negatively charged ions and then with the MS set to detect positively charged ions.

Isolation of reaction products: to isolate sufficient quantities of the DON reaction products for further characterization, additional parallel reactions were run for 72 h. In this case, concentrations were as above, but the volume of each reaction was tripled; i.e., 90 µL of DON (5 mg/mL), 450 µL of TEMPO (6.5 mM) in McIlvaine buffer at pH 5.0, and 60 µL of laccase (10 mg/mL). The dried residues of reaction mixtures were dissolved in methanol and collected, leaving behind insoluble components. Constituents were separated on a column (2.54 cm diameter; 22 cm long) of silica gel (70–230 mesh, 60 Å; Sigma-Aldrich) and eluted with a mixture of dichloromethane:methanol (95:5). Fractions (15 mL) were monitored by GC/MS. Fractions 4-6 contained the DON derivative that eluted at 6.92 min and fractions 7–9 contained the DON derivative that eluted at 5.91 min. Combined fractions were further purified on a Sephadex LH-2 (Sigma) column (2.54 cm diameter, 22 cm long) eluted with methanol.

Nuclear magnetic resonance spectroscopy (NMR): purified fractions from Sephadex chromatography were dried under a stream of nitrogen and then dissolved in deuterated chloroform. The samples were analyzed by ^1^H-, ^13^C-, heteronuclear single quantum coherence, heteronuclear multiple bond correlation, correlated spectroscopy, and distortionless enhancement by polarization transfer spectroscopy using an Avance 500 spectrometer (Bruker Biospin, Billerica, MA, USA) equipped with a 5 mm broad band observe probe with z-gradient. Topspin software (version 1.3, Bruker Biospin, Billerica, MA, USA) was used to process NMR data.
